# Social dominance and feed efficiency: Genetic analysis of latency to first meal after fresh feed delivery and residual feed intake in dairy cows

**DOI:** 10.3168/jdsc.2025-0874

**Published:** 2025-10-30

**Authors:** Ligia Cavani, Faith S. Reyes, Jennifer M.C. Van Os, Kent A. Weigel, Heather M. White, Francisco Peñagaricano

**Affiliations:** Department of Animal and Dairy Sciences, University of Wisconsin–Madison, Madison, WI 53706

## Abstract

Social dominance is present in dairy production systems and affects performance, but it is difficult to measure. Latency to first meal after feed delivery may serve as a quantifiable phenotype for social dominance, given that dominant cows typically eat first. The goal of this study was to estimate the genetic variability of latency to first meal and evaluate its association with feed efficiency and feeding behavior in dairy cows. Data consisted of 6 million bunk visits from 1,770 mid-lactation Holstein cows collected from 2009 to 2024 at the University of Wisconsin–Madison using a roughage intake control system. Latency to first meal was calculated as the time it takes for a cow to access the feed following the feed delivery. The statistical model consisted of a repeatability animal model, with lactation and DIM as fixed effects, and cohort, animal, and permanent environment as random effects. Genetic parameters for latency to first meal were estimated using daily records and weekly averages. Heritability and repeatability of latency to first meal using daily records were 0.08 ± 0.01 and 0.22 ± 0.01, respectively. Heritability and repeatability of latency to first meal using weekly averages were 0.17 ± 0.03 and 0.43 ± 0.01, respectively. Genetic correlations between weekly averages of latency to first meal and DMI, milk energy, metabolic BW, and residual feed intake were −0.03 ± 0.08, −0.12 ± 0.08, 0.10 ± 0.06, and −0.08 ± 0.10, respectively. Latency to first meal was negatively correlated with number of visits at the feed bunk per day (−0.65 ± 0.06) and total duration of visits (−0.58 ± 0.05). Latency to first meal was positively correlated with duration of each visit (0.39 ± 0.07), intake per visit (0.65 ± 0.06), and feeding rate (0.51 ± 0.07). Overall, cows with longer latencies to eat had fewer but longer bunk visits, greater intake per visit, and ate at a faster rate. Latency to first meal may be a quantifiable phenotype for social dominance; it is both heritable and repeatable and is not correlated with feed efficiency.

## Highlights

• Latency to first meal is heritable and repeatable.
• Latency to first meal is not genetically correlated with feed efficiency.
• Cows with longer latencies have fewer bunk visits and eat at a faster rate.
• Latency to first meal may be a quantifiable phenotype for social dominance.

## Graphical Abstract

**Figure:**
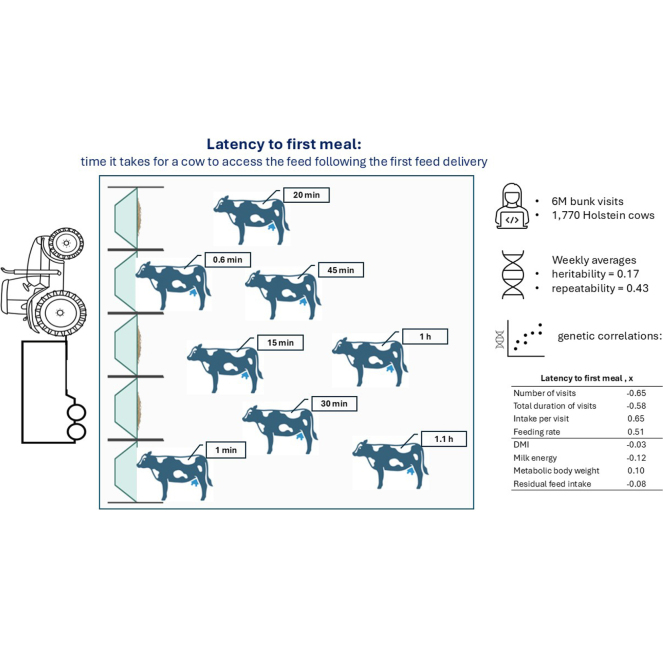
**Summary:** Social dominance in dairy cattle housed in freestall barns is mainly expressed through competitive behavior to gain access to feed. However, measuring social dominance is extremely complex, as it involves dynamic interactions among cows. There is evidence that latency to eat may serve as a quantifiable phenotype for social dominance, given that shorter latencies are associated with more dominant cows. Here, latency to first meal was calculated as the time it takes for a cow to access the feed following the first feed delivery using data from automated feeding systems, which consisted of 6 million bunk visits from 1,770 mid-lactation Holstein cows. Feeding behavior and feed efficiency were also calculated. Overall, cows with longer latencies to eat had fewer but longer bunk visits, greater intake per visit, and ate at a faster rate. Latency to first meal, based on weekly averages, is heritable (0.17) and repeatable (0.43), and it is not correlated with feed efficiency. DMI = dry matter intake.

Feed efficiency in dairy cattle has been largely studied due to its impact in farm profitability and environmental sustainability. Residual feed intake (**RFI**) is commonly used as a measure of feed efficiency, and in lactating cows, it is obtained as the difference between actual DMI and predictions from a regression on milk energy, metabolic BW, change in BW, and the effects of parity, DIM, and cohort (Berry and Crowley, 2013; Tempelman et al., 2015; VandeHaar et al., 2016). Although these models explain a large portion of the variation in DMI among mid-lactation Holstein cows, part still remains unexplained, which could help to better understand why some cows are more feed efficient than others (Cavani et al., 2023).

Genetically, feed efficiency has been associated with rumination time, lying time, activity, feeding behavior, consistency in feed intake, and feeding patterns (Cavani et al., 2022, 2024, 2025; Nascimento et al., 2024). However, little is known about the relationship between social dominance and feed efficiency, mainly due to the complexity of phenotyping social dominance, which involves sequences of interactions among animals and counts of successful or unsuccessful displacements (Krahn et al., 2023). Interestingly, Reyes et al. (2023) observed substantial variation among cows in latency to their first bunk visit after daily feed delivery, noting that cows with shorter latencies were involved in more competition. The time it takes for a cow to initiate her first meal following feed delivery could be an indicator and a quantifiable phenotype of social dominance, especially after the first daily feed delivery when there is more competition for feed. The objectives of this study were to estimate genetic parameters of latency to first meal and evaluate its association with feed efficiency and feeding behavior in lactating Holstein cows.

Data were collected from 1,770 mid-lactation Holstein cows between 2009 and 2024 in a freestall facility at the University of Wisconsin–Madison Emmons Blaine Dairy Cattle Research Center (Arlington, WI). All procedures were approved by the University of Wisconsin–Madison College of Agricultural and Life Sciences Animal Care and Use Committee. Cows were fed using a roughage intake control system (**RIC**; Hokofarm Group). The RIC system permits one animal to access the feeder at a given time and records the start and end time of each visit to the feeder, along with the amount of feed consumed during each visit. Cows (maximum of 64 per pen, with an average of 47 ± 10.7) typically had access to all 32 feeders, but in some specific experiments with dietary treatments, an individual cow may have had access to 8 or 16 randomly assigned feeders. The raw data generated by this system consisted of 6 million feeder visits and included the date, times entering and exiting the feeder, weight of feed at entry, weight of feed at exit, and the identification numbers of the transponder, cow, and feeder. For those cows that participated in multiple research trials, only data from the first trial were used. Records from feeder visits with as-fed intake ≤0 or >20 kg or visit duration <5 s or >3,000 s were removed. Using data generated by this feeding system, we calculated latency to first meal and feeding behavior traits.

Latency to first meal was calculated as the time it takes for a cow to access the feed following the first feed delivery. Cows were fed twice a day, typically at 1100 ± 1 h and 1700 ± 1 h. We were able to determine the time of first feeding precisely because the cows were locked away from the bins while farm staff discarded orts and distributed fresh feed. For the first visit after the first feed delivery, the feed weight change is negative, allowing us to identify the time of first feed delivery for each day using only data from the RIC system. Latency to first meal after the first feed delivery of the day was calculated using daily records and weekly averages (8 wk on average per cow).

The following feeding behavior traits were calculated as weekly averages: number of feeder visits per day, duration of each feeder visit, total duration of feeder visits per day, intake per visit (kg of DM), and feeding rate per visit (kg of DM/min). In addition, we calculated the intake and duration of the first feed visit following the first feed delivery.

For feed efficiency and production, the following traits were considered: DMI, secreted milk energy (**MilkE**), metabolic BW (**mBW**), and RFI. Milk samples were obtained weekly for determination of milk composition. Milk energy was calculated using the following equation (NASEM, 2021):

MilkE = (0.0929 × fat % + 0.0563 × protein % + 0.0395 × lactose %) × milk yield.

Body weights were obtained on 3 consecutive days at the beginning, middle, and end of the experimental period. We used linear regression to estimate weekly BW. Metabolic BW was calculated as the weekly average BW^0.75^, and change in BW was calculated as the difference in predicted BW at the end and beginning of each week.

Weekly RFI values were calculated using a linear mixed model as follows:

DMI = DIM + Lact + *b*_1_MilkE + *b*_2_mBW + *b*_3_ΔBW + cohort + week + e,

where DIM represents the effect of DIM with 10 levels (grouped by 15 d: 50–64, 65–79, 80–94, 95–109, 110–124, 125–139, 140–154, 155–169, 170–184, 185–200), Lact represents the effect of lactation number with 4 levels (lactation 1, lactation 2, lactation 3, and lactation 4+), MilkE is milk energy with partial regression coefficient *b*_1_, mBW is metabolic BW with partial regression coefficient *b*_2_, ΔBW is the change in BW with partial regression coefficient *b*_3_, cohort represents the random effect of experiment-treatment with 63 levels, week represents the random effect of the week of the experiment, and e is the random residual of the model, representing RFI. Random effects were assumed to follow a multivariate normal distribution, with cohort $∼N\left(0,I\sigma_{\mathrm{cohort}}^{2}\right),$ week $∼N\left(0,I\sigma_{\mathrm{week}}^{2}\right),$ e $∼N\left(0,I\sigma_{e}^{2}\right),$ and covariances between these effects equal to zero, where **I** is the identity matrix and σ^2^ represents the variance of the respective effect. Descriptive statistics for all traits are shown in Table 1.

**Table 1:** Descriptive statistics for latency to first meal after fresh feed delivery, feeding behavior, feed efficiency, and production traits in lactating Holstein cows

Trait^1^	N cows	Mean	SD	Minimum	Maximum
Latency to first meal (min)	1,770	21.4	22.7	0	218
Number of feeder visits per day	1,770	35.2	13.4	6.00	146
Duration of each feeder visit (min)	1,770	6.88	2.27	0.98	21.8
Total duration of feeder visits per day (min)	1,770	215	46.5	55.3	678
Intake per visit (kg of DM)	1,770	0.91	0.38	0.16	3.95
Feeding rate per visit (kg of DM per min)	1,770	0.15	0.05	0.05	0.70
Duration of the first feed visit (min)	1,770	5.98	3.79	0.10	32.7
Intake of the first feed visit (kg of DM)	1,770	1.13	0.70	0.02	5.62
DMI (kg/d)	1,770	27.5	4.17	12.6	42.8
Secreted milk energy (Mcal/d)	1,770	32	5.77	11.3	51.2
Metabolic body weight (kg^0.75^)	1,770	134	10.7	102	171
RFI (kg/d)	1,770	0	1.98	−11.2	12.7

1 RFI = residual feed intake.

Estimates of heritability and repeatability for latency to first meal on a daily and weekly basis were obtained using the following repeatability animal model:

**y** = **Xβ** + **Z_1_c** + **Z_2_u** + **Wpe** + **e**,

where **y** is a vector of phenotypic records, **β** is a vector of fixed effects, **c** is a vector of random cohort effects of experiment-treatment (63 levels), **u** is a vector of random additive genetic effects, **pe** is a vector of random permanent environmental effects, and **e** is the vector of random residual effects. Fixed effects included lactation number with 4 levels (1 to 4+) and DIM with 10 levels (grouped by 15 d). Matrices **X**, **Z_1_**, **Z_2_**, and **W** are incidence matrices relating **y** to **β**, **c**, **u**, and **pe**, respectively. Random effects were assumed to follow a multivariate normal distribution,

$$\left(\begin{array}{c} c\\ u\\ \mathrm{pe}\\ e \end{array}\right)\&sim;N\left(\left(\begin{array}{c} 0\\ 0\\ 0\\ 0 \end{array}\right),\;\left(\begin{array}{cccc} I\sigma_{c}^{2} & 0 & 0 & 0\\ 0 & A\sigma_{u}^{2} & 0 & 0\\ 0 & 0 & I\sigma_{\mathrm{pe}}^{2} & 0\\ 0 & 0 & 0 & I\sigma_{e}^{2} \end{array}\right)\right),$$

where $\sigma_{c}^{2},$ $\sigma_{u}^{2},$ $\sigma_{\mathrm{pe}}^{2},$ and $\sigma_{e}^{2}$ are the cohort, additive genetic, permanent environmental, and residual variances, respectively; **I** is the identity matrix; and **A** is the matrix of additive relationships between animals in the pedigree using the last 3 generations.

Estimates of genetic correlations between latency to first meal and feeding behavior, feed efficiency, and production traits, calculated as weekly averages, were performed in bivariate analyses using a repeatability animal model. Bivariate models included the same fixed effects as the univariate models, and random effects were assumed to follow a multivariate normal distribution,

$$\left(\begin{array}{c} c\\ u\\ \mathrm{pe}\\ e \end{array}\right)\&sim;N\left(\left(\begin{array}{c} 0\\ 0\\ 0\\ 0 \end{array}\right),\;\left(\begin{array}{cccc} C_{0}⊗I & 0 & 0 & 0\\ 0 & G_{0}⊗A & 0 & 0\\ 0 & 0 & P_{0}⊗I & 0\\ 0 & 0 & 0 & R_{0}⊗I \end{array}\right)\right),$$

where **C_0_**, **G_0_**, and **P_0_** are the 2 × 2 cohort, additive genetic direct, and environmental permanent effects (co)variance matrices, respectively; **A** is the matrix of additive relationships between animals in the pedigree of the last 3 generations; **R_0_** is the 2 × 2 residual (co)variance matrix; and **I** is an identity matrix with suitable dimensions. For RFI, only the random additive genetic effects and the random permanent environmental effects were considered, as the other effects had already been considered in the calculation of the RFI phenotype.

Estimates of heritability, repeatability and genetic correlations were obtained using restricted maximum likelihood method through the blupf90+ software from the BLUPF90 family of programs (Aguilar et al., 2018).

Relationships between latency to first meal and the traits currently considered in the US national genetic evaluations of dairy cattle, namely production, longevity, health, reproduction, and efficiency, were analyzed using Spearman's rank correlations between sires' breeding values. The sires' breeding values were provided by the Council on Dairy Cattle Breeding from the official evaluation (December 2024). Only sires with records from at least 10 daughters were considered, resulting in a subset of 110 sires.

On average, cows took 21 min to start their first meal after first feed delivery, which is consistent with the findings of a trial at the same facility with 64 cows, where cameras were used to determine when feed was delivered (Reyes et al., 2024). This shows that the method we developed is reliable, making it possible to automate the phenotyping for traits related to first feed delivery, such as latency, with no need for cameras. It can also be applied to historical data from automated feeding systems. It should be noted that our findings were obtained under a competitive 2:1 feed bunk stocking density, which may not fully translate to open feed bunk systems, where latency averages could differ under those conditions.

Estimates of heritability and repeatability for daily latency to first meal were 0.08 ± 0.01 and 0.22 ± 0.01, respectively. When latency to first meal was aggregated into weekly averages, the heritability and repeatability estimates increased to 0.17 ± 0.03 and 0.43 ± 0.01, respectively.

Higher estimates for weekly averages are expected, as averaging daily records reduces residual variance. Latency to first meal is heritable and repeatable across weeks, indicating that a proportion of the phenotypic variation is explained by additive genetic effects and that it could therefore be incorporated into genetic selection schemes. To our knowledge, this is the first study to estimate the heritability and repeatability of latency to first meal.

Genetic correlation estimates between latency to first meal and feed efficiency and production, as well as feeding behavior traits, measured as weekly averages, are depicted in Figure 1. Latency to first meal was not genetically correlated with RFI (−0.08 ± 0.10), DMI (−0.03 ± 0.08), milk energy (−0.12 ± 0.08), or metabolic BW (0.10 ± 0.06). These results suggest that genetic selection for feed efficiency may not affect the time cows take to start their first meal. They also indicate that feed efficiency in lactating Holstein cows might not be associated with certain strategies relating to competition for food. Nevertheless, latency to first meal was genetically correlated with feeding behavior. Longer latency to first meal was genetically associated with less time spent at the bunk per day (−0.58 ± 0.05) and fewer bunk visits (−0.65 ± 0.06), but with longer duration per visit (0.39 ± 0.07), greater intake per visit (0.65 ± 0.06), and a faster eating rate per visit (0.51 ± 0.07). Stock density is an important factor in behavioral changes, where greater animal density is associated with more competition among cows and could cause a decrease in intake for nondominant cows (DeVries et al., 2004). Our findings showed that in a less competitive situation, less dominant cows eat later (longer latency to first meal), visit the feed bunk less frequently to avoid competition, but eat faster to compensate and maintain their daily intake. In one of our previous studies, a faster eating rate was associated with poorer feed efficiency (Cavani et al., 2022). The fact that latency to the first meal after feed delivery was not associated with feed efficiency in the current study, but was associated with feeding rate, might suggest that cows with shorter latency are able to sort the TMR, which reduces the quality of feed available for cows with longer latency. Moreover, Spearman's rank correlations of sires' breeding values showed no important associations between latency to first meal and 22 different traits currently considered in the US national genetic evaluation, grouped as production, longevity, health, reproduction, and feed efficiency traits (Table 2). The only significant Spearman's rank correlation (*P*-value <0.05) was with milk fever resistance (−0.26), showing a possible weak association between longer latency to first meal and lower resistance to milk fever.

**Figure 1:**
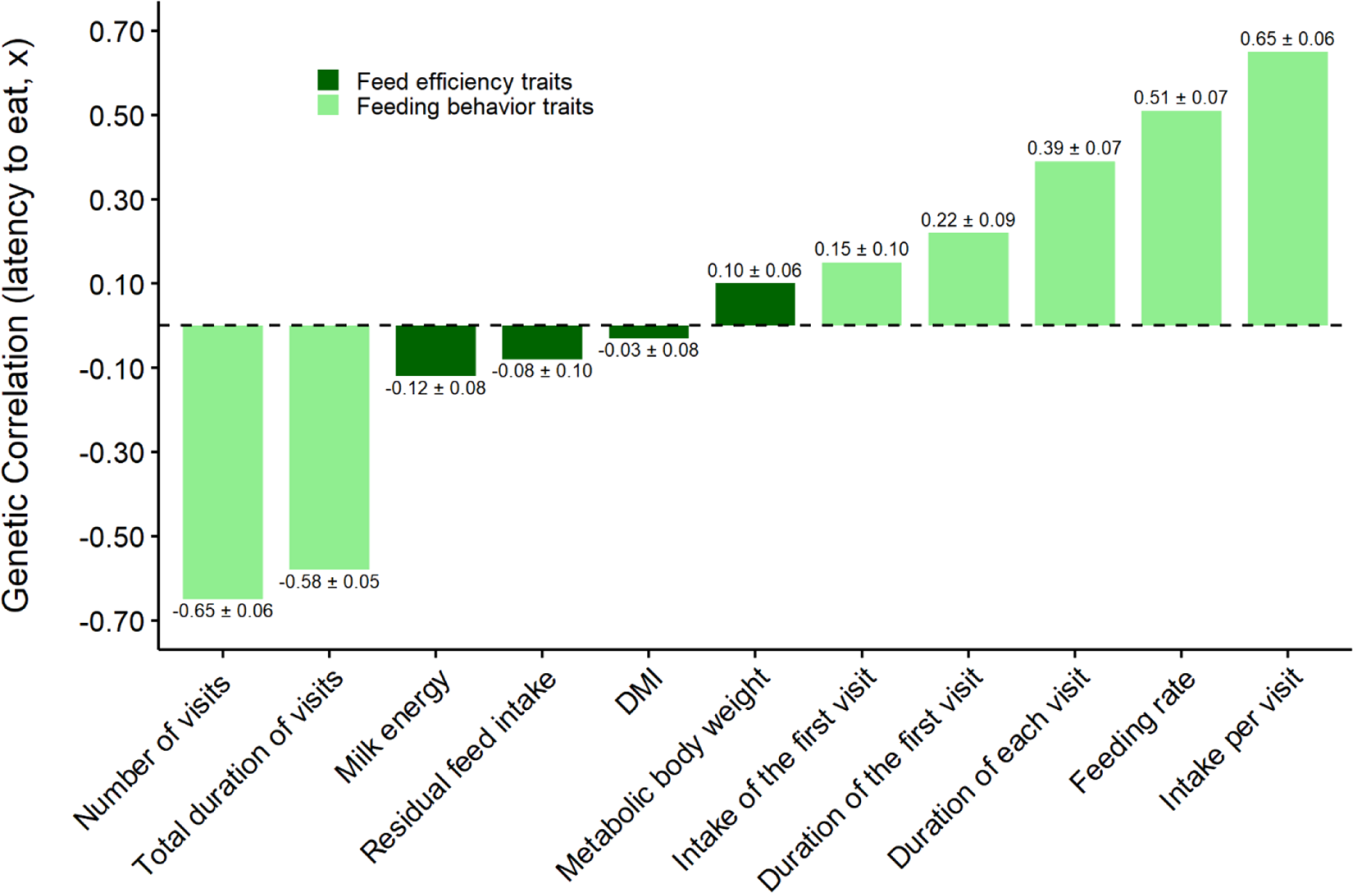
Estimates of genetic correlations (±SE) between latency to first meal after fresh feed delivery and feeding behavior, production, and feed efficiency traits in lactating Holstein cows.

**Table 2:** Relationships between latency to first meal after fresh feed delivery and traits considered in the US national genetic evaluations of dairy cattle assessed using Spearman's rank correlations of breeding values of 110 sires with records from at least 10 daughters

CDCB genetic evaluation trait^1^	Spearman's rank correlation with latency to first meal
Production	
Milk yield	−0.06
Fat yield	−0.04
Fat %	0.01
Protein yield	−0.06
Protein %	−0.02
Longevity	
Productive life	0.05
Cow livability	−0.10
Heifer livability	0.01
Gestation length	0.08
Health	
Somatic cell score	0.04
Milk fever resistance	−0.26
Displaced abomasum resistance	0.07
Ketosis resistance	0.11
Mastitis resistance	−0.14
Metritis resistance	0.12
Retained placenta resistance	0.08
Reproduction	
Cow conception rate	−0.02
Heifer conception rate	0.20
Daughter pregnancy rate	−0.01
Early first calving	0.01
Feed efficiency	
Feed saved	0.01
Residual feed intake	−0.05

1 CDCB = Council on Dairy Cattle Breeding.

Social dominance in dairy cattle housed in freestall systems is mainly expressed through competitive behavior to gain access to feed (Val-Laillet et al., 2008). This raises the hypothesis that it may be associated with feed efficiency. However, measuring social dominance is extremely complex, as it involves dynamic interactions among cows and is related to group characteristics. This is a limitation, especially in genetics studies, due to the challenge of large-scale phenotyping. There is evidence that behavioral traits observed around first feed delivery could be used as an indicator of social dominance in dairy cows. For example, regardless of parity number, visiting the bunk sooner after feed delivery was associated with more competition, as measured via continuous video recordings (Reyes et al., 2023; 2024). Indeed, the results of our study showed that latency to first meal could be a quantifiable phenotype of social dominance, as it showed similar associations to those reported in other studies using more direct methods to measure social dominance. For instance, no correlation was found between RFI and any competitive behavior (Reyes et al., 2023), whereas a shorter latency was associated with more time spent eating after feed delivery (Reyes et al., 2024). Moreover, Fiol et al. (2019) did not find an association between dominant versus subordinate heifers and DMI, but they observed that subordinate heifers ate at a faster rate. Genetic selection for longer latency could favor less dominant behavior at the feed bunk without affecting feed efficiency or milk production. In addition, incorporating latency to first meal into a genetic selection scheme could help reduce competition at the bunk, which may be greater under commercial conditions, and improve overall herd welfare.

Overall, our findings suggest that cows with longer latencies to first meal after fresh feed delivery had fewer but longer bunk visits, greater intake per visit, and ate at a faster rate. This suite of behaviors may indicate distinct behavioral feeding styles whereby some cows avoid direct competition by waiting longer to access fresh feed and visiting the feed bunk less frequently, but compensate to maintain daily intake by eating faster and more at each visit. Additionally, our results showed that latency to first meal is heritable and repeatable across weeks, and it is not correlated with feed efficiency in lactating Holstein cows.
